# Harnessing filamentous phages for enhanced stroke recovery

**DOI:** 10.3389/fimmu.2023.1343788

**Published:** 2024-01-16

**Authors:** Yang Li, Kai-di Yang, De-cai Kong, Xiao-meng Li, Hao-yu Duan, Jun-feng Ye

**Affiliations:** ^1^ General Surgery Center, First Hospital of Jilin University, Changchun, Jilin, China; ^2^ School of Nursing, Jilin University, Changchun, China

**Keywords:** stroke, filamentous phages, bacteriophage therapy, neurological restoration, tissue regeneration

## Abstract

Stroke poses a critical global health challenge, leading to substantial morbidity and mortality. Existing treatments often miss vital timeframes and encounter limitations due to adverse effects, prompting the pursuit of innovative approaches to restore compromised brain function. This review explores the potential of filamentous phages in enhancing stroke recovery. Initially antimicrobial-centric, bacteriophage therapy has evolved into a regenerative solution. We explore the diverse role of filamentous phages in post-stroke neurological restoration, emphasizing their ability to integrate peptides into phage coat proteins, thereby facilitating recovery. Experimental evidence supports their efficacy in alleviating post-stroke complications, immune modulation, and tissue regeneration. However, rigorous clinical validation is essential to address challenges like dosing and administration routes. Additionally, genetic modification enhances their potential as injectable biomaterials for complex brain tissue issues. This review emphasizes innovative strategies and the capacity of filamentous phages to contribute to enhanced stroke recovery, as opposed to serving as standalone treatment, particularly in addressing stroke-induced brain tissue damage.

## Introduction

Stroke, an acute cerebrovascular condition that leads to cerebral tissue injury, ranks as the second leading cause of mortality worldwide and the third leading cause of disability. It accounts for over ten million cases annually, with ischemic strokes being predominant (1). The onset of a stroke is primarily attributed to cerebral ischemia or hemorrhage resulting from vascular blockage or rupture (2). Existing treatments rely on timely detection and intervention. Nevertheless, many cases fail to meet this critical timeframe. Moreover, conventional therapies are constrained by various limitations and adverse effects (3). To address these challenges, there is a growing momentum in the search for pioneering medical strategies aimed at restoring compromised brain function (4). Current interventions for acute stroke encompass hyperbaric oxygen therapy, rehabilitative training, and the administration of neurotrophic agents or anti-inflammatory compounds (5) (**Table 1**). However, these treatments are marred by complications such as oxygen toxicity, barotrauma, and decompression sickness, and their effectiveness in facilitating brain recovery is limited. In the context of post-stroke cerebral tissue rehabilitation, the establishment of a robust vascular network (angiogenesis) emerges as a pivotal factor. This process contributes to the restoration of both endogenous and transplanted neural stem cells (NSCs) and the regeneration of neuronal networks (neurogenesis) (6). However, achieving angiogenesis and neurogenesis in stroke potential therapy for brain regeneration remains a significant challenge. Although specific biomaterials like poly(lactic-co-glycolic acid) (PLGA) microparticles, hyaluronic acid hydrogels, and polymer scaffolds show potential in promoting angiogenesis or neurogenesis in stroke-affected areas, their overall effectiveness remains limited (7). For instance, an injectable dual-function angiogenic hydrogel, incorporating vascular endothelial growth factor but lacking neural stem cells (NSCs), has demonstrated the ability to induce angiogenesis and neuronal growth (8). However, this hydrogel’s potential in fostering extensive axonal growth within the cavity center was hampered due to challenges in cellular infiltration within the compromised brain tissue. Neuronal revival predominantly relied on the migration and differentiation of neuroblasts from the subventricular zone (SVZ). Nonetheless, temporal constraints on neuroblast proliferation within the SVZ and the spatial gap between the SVZ and the stroke lesion limited the effectiveness of this approach (9). Consequently, the demand for novel multifunctional materials becomes evident as a means to achieve more efficacious clinical treatments (10).

**Table 1:** Drugs for treating stroke.

Drug class	Drug
Improving blood circulation	
Antithrombotic	Heparin,vitamin K antagonists, tirofiban
Anti-platelet	Aspirin,clopidogrel
Thrombolytics	Alteplase (FDA's exclusive approval), Tenecteplase, Non-immunogenic recombinant staphylokinase, Recombinant human prourokinase
Fibrinogen-depleting agents^1^	Maraviroc
Neuroprotective drugs	
Calcium channel blockers^2^	Amlodipine
Antioxidant and nitrous stress	NYP-059
γ-aminobutyric receptor^3^ agonists agonists	Diazepam and Clomethiazole
Opioid antagonists^4^	Naloxone, Nalmefene
NMDA channel blockers^5^	Dextrorphan
AMPA channel blockers	Perampanel
Stroke recovery-promoting	
Selective Serotonin Reuptake Inhibitors SSRI^6^	Fluoxetine
Neurotrophic factors^7^	Stem cell factor, Granulocyte-colony stimulating factor

^1^The principal mechanism of action for fibrinogen-depleting agents lies in their capacity to trigger the conversion of fibrinogen within thrombi into fibrinolytic enzymes, facilitating the breakdown of fibrin aggregates and gradual dissolution of the thrombus. This process aids in the restoration of unobstructed vascular flow, thereby reinstating cerebral blood supply and mitigating or circumventing further neural tissue damage. ^2^Primarily employed in cardiovascular disease management, calcium channel blockers may confer therapeutic benefits in post-stroke recovery owing to their distinct mode of action. By impeding calcium ion influx into cells, these agents diminish neuronal injury, while inhibiting smooth muscle calcium entry results in vasodilation. Furthermore, their impact on neurotransmitter release contributes to neural functional recuperation post-stroke. ^3^Gamma-aminobutyric receptor agonists not only possess neuroprotective attributes but also hold promise in augmenting post-stroke recovery. ^4^The prolonged use of opioid medications heightens stroke risk and severity. Opioid receptor antagonists, by mitigating blood-brain barrier damage, exhibit potential in lessening stroke severity and promoting neural recovery. ^5^NMDA, pivotal in stroke-induced excitotoxicity, is targeted by NMDA antagonists to curb excessive excitation, thereby affording neuronal protection. ^6^Selective serotonin reuptake inhibitors (SSRIs) demonstrate the ability to facilitate neural recovery in stroke patients. They foster hippocampal neurogenesis and neurotrophic factor secretion, influencing the balance of excitatory and inhibitory elements within the brain. ^7^Neurotrophic factors activate a myriad of growth processes, encompassing neurogenesis, angiogenesis, axonogenesis, and myelination. Beyond this, they play a vital role in safeguarding neurons and improving behavioral outcomes, manifesting their significance in neural recovery and protection.

The formation of a cavity in brain tissue following a stroke stems from autophagy-induced degradation of the affected region, resulting in the absence of the usual extracellular matrix that supports cellular infiltration and tissue regeneration within the cavity (11). This distinctive cavity presents an ideal location for transplantation in stroke potential therapy (12). A nascent avenue, phage regenerative therapy harnesses phage attributes to facilitate tissue revitalization (13). Bacteriophages, nanostructured viruses capable of infecting and eradicating specific bacteria, have been employed to combat refractory bacterial infections (14) and offer a wide array of applications in the field of biological nanomaterials, presenting themselves as potential alternatives to antibiotics (15). The fusion of biology and materials science through phage display has been instrumental in propelling the advancement of biogenic nanomaterials-a pioneering concept first introduced by Nobel laureate George Smith in 1985 (16). This approach capitalizes on the diversity of phages, resulting in expansive libraries housing numerous clones with distinct peptides, facilitating the precise selection of receptors and enzymes (17). Recent insights extend beyond infection control, revealing bacteriophages’ impact on host immune and metabolic systems, contributing to the mitigation of pathological processes associated with chronic inflammation and metabolic disorders (18). For example, a specific peptide screened from a bacteriophage library, when conjugated to nano-complexes, exhibits optimal inhibition of activated hepatic stellate cell migration and achieves the highest uptake in rat livers, serving as an effective platform for alleviating liver fibrosis (19, 20). These revelations provide both theoretical underpinnings and empirical groundwork for the application of filamentous phage regenerative therapy. Its potential application in enhancing stroke recovery takes center stage, addressing a prevailing neurological disorder stemming from cerebral vascular blockages or ruptures, which induce ischemia and hypoxia. Stroke-triggered brain tissue experiences necrosis, apoptosis, autophagy, and inflammatory reactions that exacerbate damage (21). Filamentous phage regenerative therapy presents a promising pathway for enhancing stroke recovery, unveiling novel mechanisms for safeguarding and restoring post-stroke brain tissue. Bacteriophages showcase varied capabilities, spanning pathogen clearance, immune modulation, promotion of neurogenesis, and enhancement of vascular function (22–24). These combined effects culminate in neuroprotection, diminished edema, reduction in infarct size, and improved neural functionality. Despite its promise, the realm of filamentous phage regenerative therapy is still in its infancy, largely investigated in animal models, yet to undergo clinical trials. Aspects concerning safety, effectiveness, dosage, delivery, and timing demand additional investigation and validation. Challenges encompass selectivity, stability, tolerability, and resistance. Thus, the establishment of the potential therapy’s efficacy in stroke management necessitates meticulous scientific substantiation and technical reinforcement to create a dependable and efficacious treatment approach.

Recent investigations have illuminated the central role of regulatory T cells (Tregs) in orchestrating immune modulation and tissue restoration, infiltrating the aftermath of stroke and rousing microglia via osteopontin (OPN) secretion, thereby igniting the generation of oligodendrocyte progenitor cells (OPCs) and the rejuvenation of myelin sheaths (25). Experimental indications underscore that filamentous phages, operating via these intricate genetic modifications, strengthen neurological function and post-stroke prognosis. The modified filamentous bacteriophage promotes the generation of oligodendrocyte precursor cells, stimulates myelin regeneration, significantly enhances neural function and cognitive abilities, and facilitates neural repair. Additionally, it serves as a potential alternative to autologous nerve transplants for restoring extensive nerve damage (22, 26). These investigations not only underscore the promising potential of phage therapy in the context of enhancing stroke recovery but also emphasize the need for meticulous parameter optimization encompassing aspects like selection, dosing, administration routes, and temporal considerations to ensure heightened safety and efficacy. Furthermore, the attainment of comprehensive clinical trials remains imperative for the validation of its effects within human subjects.

**Figure 1:**
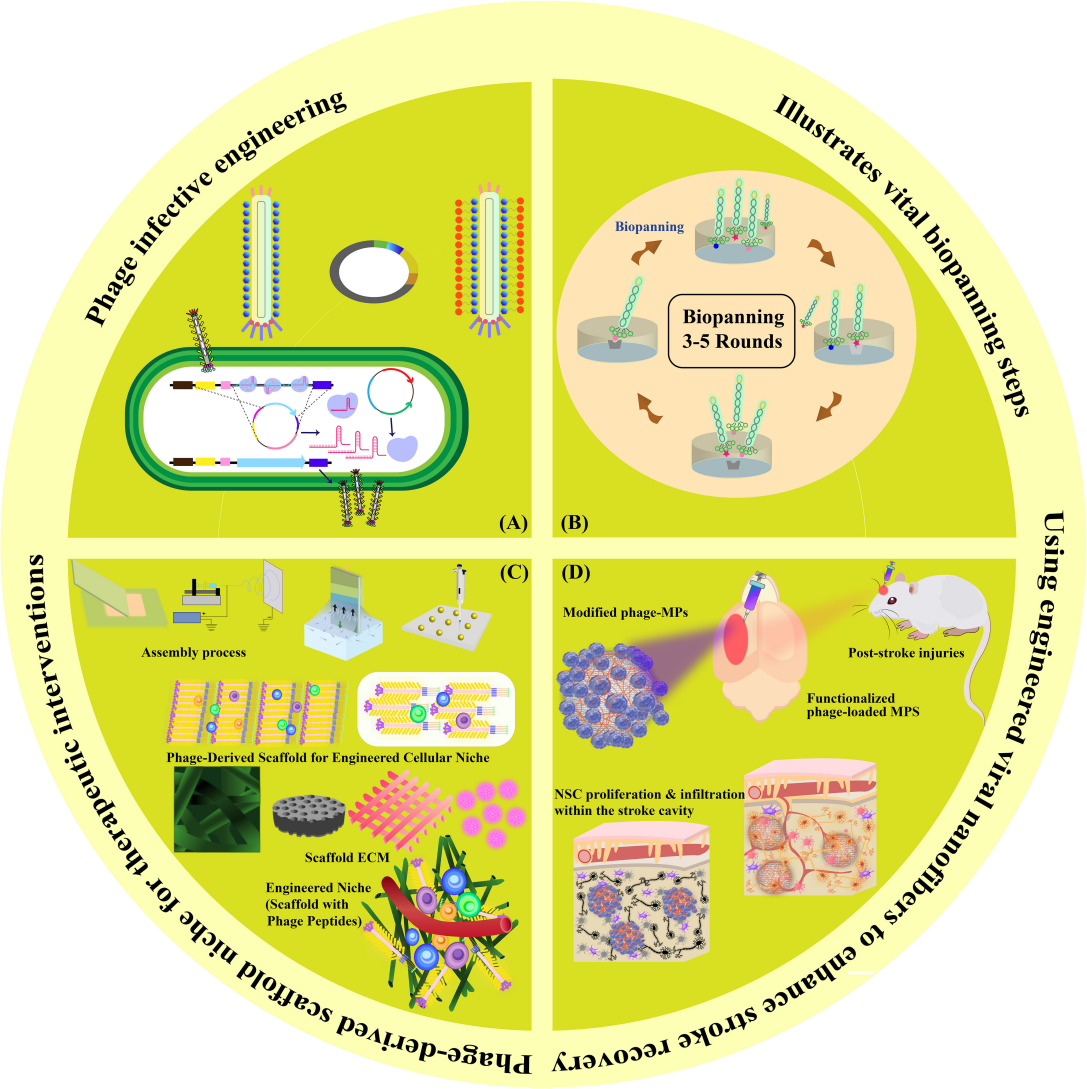
Graphical Abstract. This visual summary provides an overview of the multifaceted mechanisms explored in our review for *in situ* tissue regeneration in stroke potential therapy. The depiction encapsulates the sequential exploration of phage infective engineering, biopanning techniques in phage display, phage-derived scaffolds for cellular niches, engineered niche scaffolds with phage peptides, and functionalized phage-loaded MPS for NSC proliferation and infiltration within the stroke cavity.

In this review, we navigate through the multifaceted landscape of mechanisms employed in *in situ* tissue regeneration for stroke potential therapy. We commence by investigating the foundational principles of filamentous phage infective engineering and explore its applications in stroke therapy. Next, we dive into the biopanning techniques applied within the phage display framework, highlighting their significance in identifying therapeutic targets. Our journey continues with an examination of the pioneering utilization of filamentous phage-derived scaffolds for engineering cellular niches, shedding light on their role in fostering tissue regeneration. We delve deeper into the creation of engineered niche scaffolds, enriched with phage peptides, revealing their unique potential in enhancing post-stroke recovery. With this structured approach, we aim to provide a comprehensive insight into the potential of harnessing these mechanisms for enhancing stroke recovery, offering a detailed account of each element’s contribution to the field (**Figure 1**).

## Filamentous bacteriophage potential therapy for stroke recovery

### The role of filamentous phages in therapeutics

Filamentous bacteriophages, such as fd, f1, and M13, belong to the Inovirus genus and possess a unique nanofiber-like structure. Among the various phage categories, filamentous phages, particularly M13 phages, emerge as widely adopted vectors for both display purposes and bionanomaterial manufacturing (27–31). M13 phages possess a semi-flexible, nanofiber-like structure, adept at self-assembling into diverse ordered forms responsive to their surroundings (17, 30, 32–35). Studies employing nuclear magnetic resonance spectroscopy have uncovered that both M13 and fd phages maintain highly conserved and resilient structures, capable of enduring severe conditions, with M13 phages displaying slightly augmented rigidity (36). Phages are generally categorized into various types, such as lytic phages and temperate phages. In contrast to the commonly observed lytic tailed phages of the Caudovirales family (e.g., T1, T4, T7) used in phage therapy, M13 phages exhibit lysogenic behavior, establishing persistent infections within their host without causing lytic destruction. Leveraging these characteristics, M13 phages have emerged as a preferred asset for various bionanomaterial applications (37–39). Its capability to host multiple peptides on a single phage, achieved through the insertion of foreign DNA fragments into filamentous phage gene III, offers a valuable characteristic for contemporary multifunctional nanomaterials, allowing precise protein modification and multi-peptide presentation (40, 41). Virion capsids, such as M13 phages, provide robust stability for drug delivery, enhancing precision and therapeutic potential. Abundant viral-like particles (VLPs) like M13 phages offer cost-effective reservoirs for tailored drug packaging and precise targeting in nanocarrier-based drug delivery systems, ensuring biocompatibility and uptake efficiency (30, 33, 34, 42).

### Enhancing therapeutics through modification

**Figure 2:**
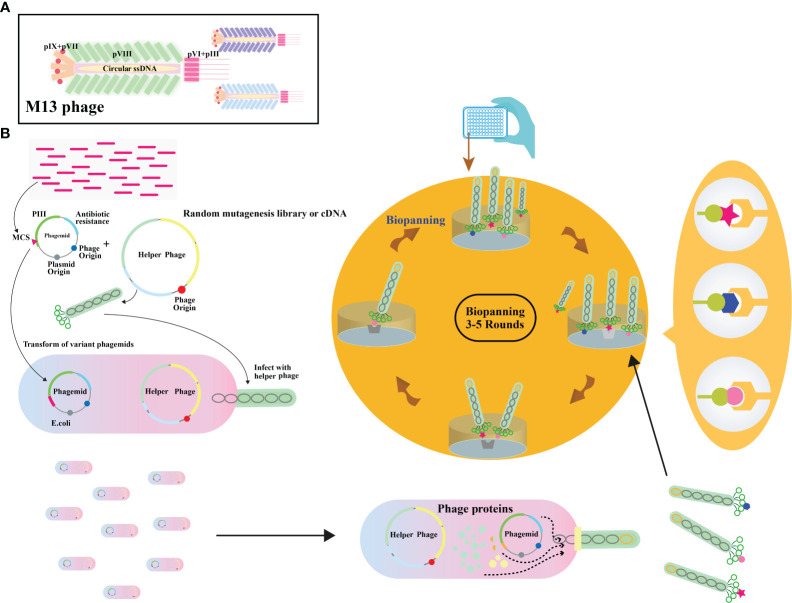
**(A)** Illustration depicting the overall architecture of the M13 phage. **(B)** Schematic representation of the phage display biopanning process. A DNA library, either natural or synthetic, is inserted between the signal peptide and pIII encoding gene on a phagemid vector. The phagemid pool is introduced into Escherichia coli cells already infected with a helper phage. A subset of these capsids carries the inserted peptides. The phage library then undergoes iterative rounds of biopanning. Adapted from (64).

**Figure 3:**
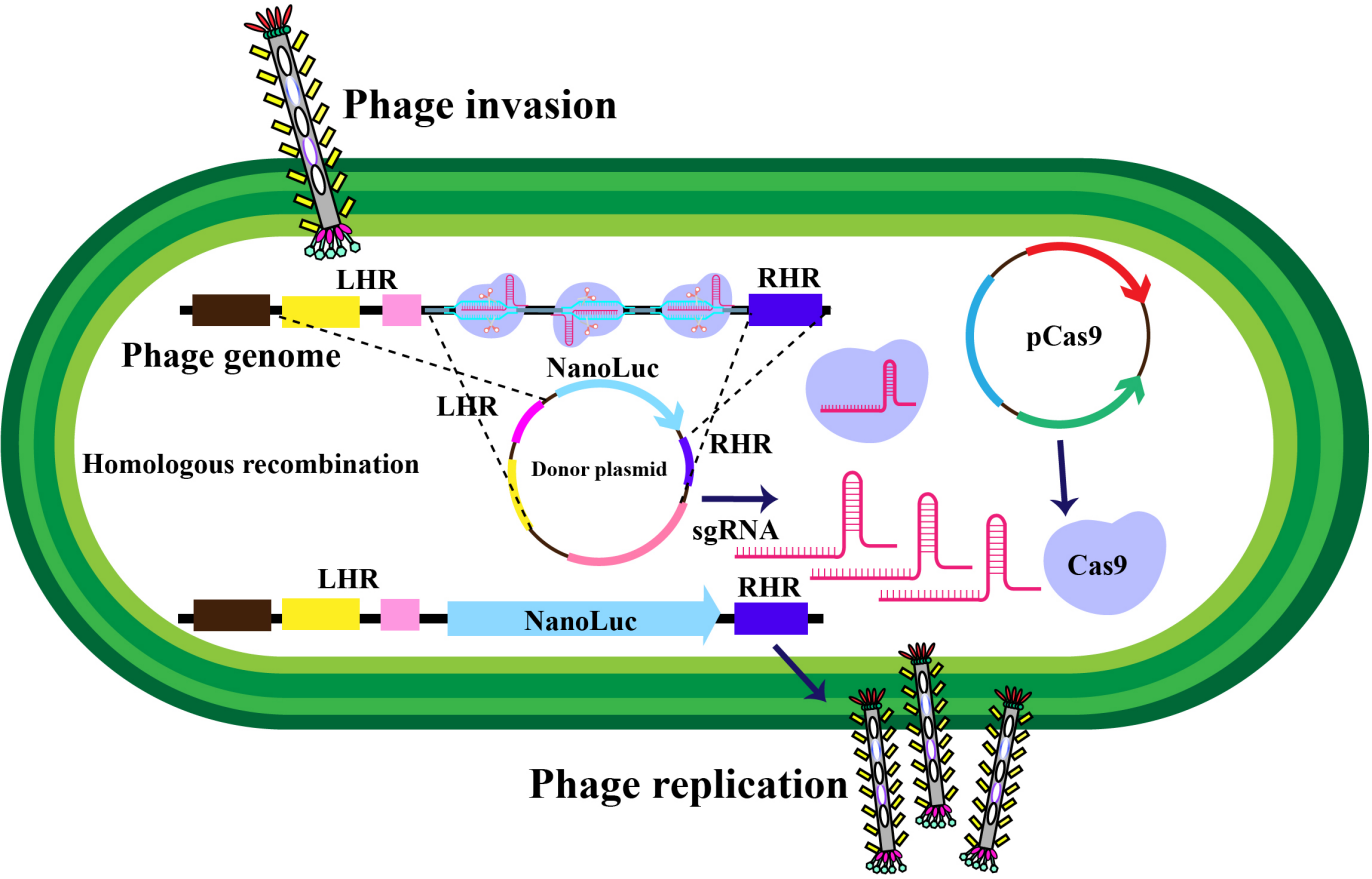
Schematic representation of *in-vivo* phage recombination facilitated by the CRISPR-Cas9 system. Host bacterial cells undergo transformation with a donor plasmid harboring the NanoLuc reporter gene flanked by left and right homology regions to the phage, alongside specifically chosen CRISPR RNA and genome-targeting sequences. Adapted from (65).

Filamentous phages, notably M13 phages, present a promising trajectory in therapeutics owing to their distinctive nanofiber-like structure. Through genetic modifications, these phages enable a safe and stable multidimensional assembly. Presently, with the widespread application of phage therapy, the genetically engineered M13 phage, as a biological scaffold, facilitates easier purification and amplification compared to wild-type phages. Consequently, this chimeric phage-based therapy allows for the safe and effective identification and monitoring of bacteria, as well as the implementation of controlled antibacterial strategies through assembly with inorganic nanomaterials (43–46). Moreover, their genetic adaptability allows for precise and personalized medicine (47). The utilization of genetically engineered filamentous phages as bionanomaterials unlocks exciting opportunities for targeted cancer therapy, tissue regeneration, and drug delivery. These applications directly address the scarcity of tissue and organ donors, establishing these phages as creative resources in the field of nanomedicine (48). The filamentous bacteriophages stimulate the proliferation and differentiation of neural stem cells, fostering the regeneration of neurons and glial cells (49). In addition, they elevate cerebral blood flow, vascular function, neovascularization, and angiogenesis, collectively contributing to improved neurovascular outcomes (49). These diverse actions position bacteriophages as potential therapeutic agents to protect neurons, mitigate brain edema, minimize infarct size, and boost neurological function (**Table 2**). Phage display’s utility extends to developing peptides for drug transport across the blood-cerebrospinal fluid barrier (62). M13, fd, and f1 phages, with their versatile coat proteins, are prominent vectors for diverse applications (63). **Figures 2A, B** illustrates vital biopanning steps: rinsing to remove unbound virions, selectively collecting interacting virions, and propagating phages with strong ligand affinities via *E. coli* infection. The CRISPR/Cas system can be applied to engineer bacteriophages, enabling targeted alterations and creating novel phage variants (**Figure 3**) (47, 65). Utilizing the CRISPR-Cas system for genetic manipulation of filamentous or other bacteriophages has enabled the isolation of desired phage mutants, showcasing the significant utility of CRISPR-Cas in the genetic engineering of bacteriophages (47, 66). The development of genetic engineering techniques to modify phages such as M13, T5, and T7 involves diverse applications like single-base substitutions, deletions or insertions of base pairs, and more. This versatile technique is readily adaptable for use across a spectrum of bacterial and phage strains (47, 67).

**Table 2:** Synthetic bacteriophages promote tissue regeneration (39, 50–61).

Peptide sequence	Targeting and binding	Biological activity	Potential application	Ref
RGD(Arg-Gly-Asp)	Integrin αvβ1, αvβ3, αvβ5, αvβ6, αvβ8	Cellular adhesion	Tissue engineering, Drug delivery, Angiogenesis promotion	(Yang, Zhang et al., 2021) (60); (Safari, Sadeghizadeh et al., 2022) (56)
DEGA(Asp-Gly-Glu-Ala)	Integrin α2β1	Cellular adhesion	Tissue engineering, Drug delivery	(Yoo, Kobayashi et al., 2011) (61)
IKVAV	Integrin α6β4	Cellular adhesion	Neural Regeneration, Peripheral Nerve Repair	(Merzlyak, Indrakanti et al., 2009) (39)
I75 and I105(TASNLQSQQAYAAPTT)	H3 peptide, L1CAM	Cellular adhesion	Tissue engineering, regeneration	(Tang, Yu et al., 2015) (59)
RRQTLSHQMRRP	Nogo-66	Neuron survival and axonal regeneration	Tissue engineering, regeneration	(Deng, Cai et al., 2013b) (53); (Deng, Cai et al., 2013a) (52)
NAP2(HITRALV)	Nogo-66	Neuron survival and axonal regeneration	Tissue engineering, regeneration	(Sun, Dai et al., 2016) (58)
CGLPYSSVC	netrin-4	Cellular proliferation and adhesion	Tissue engineering, regeneration	(Staquicinia, Dias-Neto et al., 2009) (57)
FAQRVPP	neural stem cell (NSC)-derived neural precursor cells (NPCs)	Cellular differentiation,	Tissue engineering, regeneration	(Gelain, Cigognini et al., 2012) (54)
BMHP1(RADARADARADARADAGGGGPFSSTKT)	Bind with NSC	cellular adhesion and proliferation	Tissue engineering, regeneration	(Cigognini, Satta et al., 2011) (51)
KLPGWSG	Bind with NSC	cellular differentiation and proliferation	Tissue engineering, regeneration	(Caprini, Silva et al., 2013) (50)

**Figure 4:**
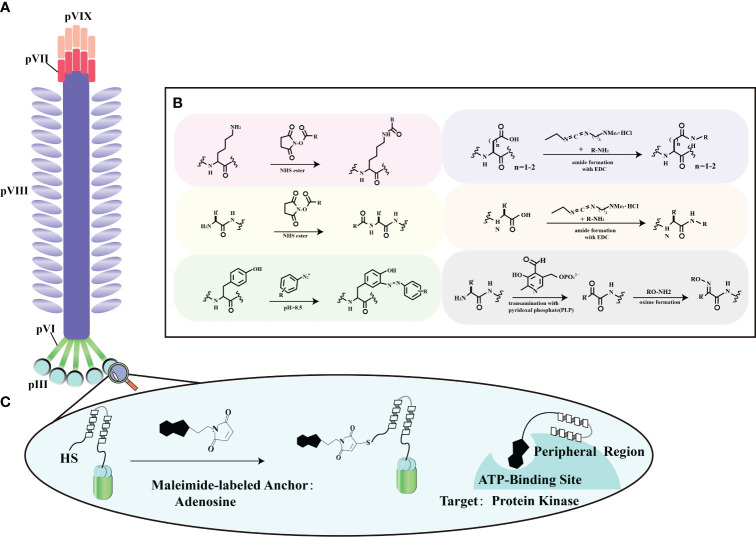
Chemical modification strategies for filamentous phage. **(A)** General locations of coat proteins. This panel illustrates the fundamental spatial distribution of coat proteins in filamentous phages, forming the basis for our chemical modification strategies. **(B)** Typical reactions for functional group modification. In this section, we outline the standard chemical reactions employed to selectively modify specific functional groups on these proteins, enhancing the adaptability of filamentous phage engineering. Adapted from (68) **(C)** Chemical modification of a phage-displayed peptide. Panel **(C)** depicts the chemical modification of a phage-displayed peptide using adenosine as an anchoring molecule. This modification is tailored to facilitate binding to the ATP binding site of protein kinases, demonstrating potential applications in advanced protein-protein interaction studies. Adapted from (69).

The surface proteins of bacteriophages offer a rich assortment of diverse amino acids, making them amenable to chemical modifications using various molecules (**Figures 4A, B**). Ongoing advancements in biotechnology now enable the *in vitro* synthesis of substantial DNA fragments, including entire bacteriophage genomes. This capability greatly facilitates the chemical alteration of bacteriophages, allowing for the creation of novel genomic sequences and streamlining genetic editing. It’s worth noting that there are some limitations when dealing with larger DNA molecules (70). Surface proteins, particularly in the case of the M13 bacteriophage, most notably pIII, serve as versatile platforms for the introduction of functional groups through chemical modifications (**Figure 4C**). Viral protein shells are chemically modified to enhance their functions or change their characteristics. Techniques include altering amino acid residues (71), natural chemical linkages (72), and incorporating non-natural amino acids (73). Eun-A. Kwak et al. showcased a method for site-specific chemical modification of fd bacteriophages using the formylglycine-generating enzyme (FGE). This enzyme selectively modifies the conserved peptide sequence CXPXR. By genetically modifying the fd bacteriophage, researchers can place the CXPXR motif at different locations, providing controlled sites for specific chemical attachment. Kwak et al. added the FGE recognition motif to the p3 protein shell, allowing site-specific enzyme-catalyzed modification to attach the bacteriophage to various surfaces with amino functional groups. This method introduces a new way of modifying viruses, affirming efficient viral engineering techniques by coupling them selectively to diverse chemical targets (74). This fine-tuning optimizes surface properties, supporting targeted therapeutic engineering in enhancing stroke recovery.

### Harnessing mechanisms of *in situ* tissue regeneration for stroke recovery

**Figure 5:**
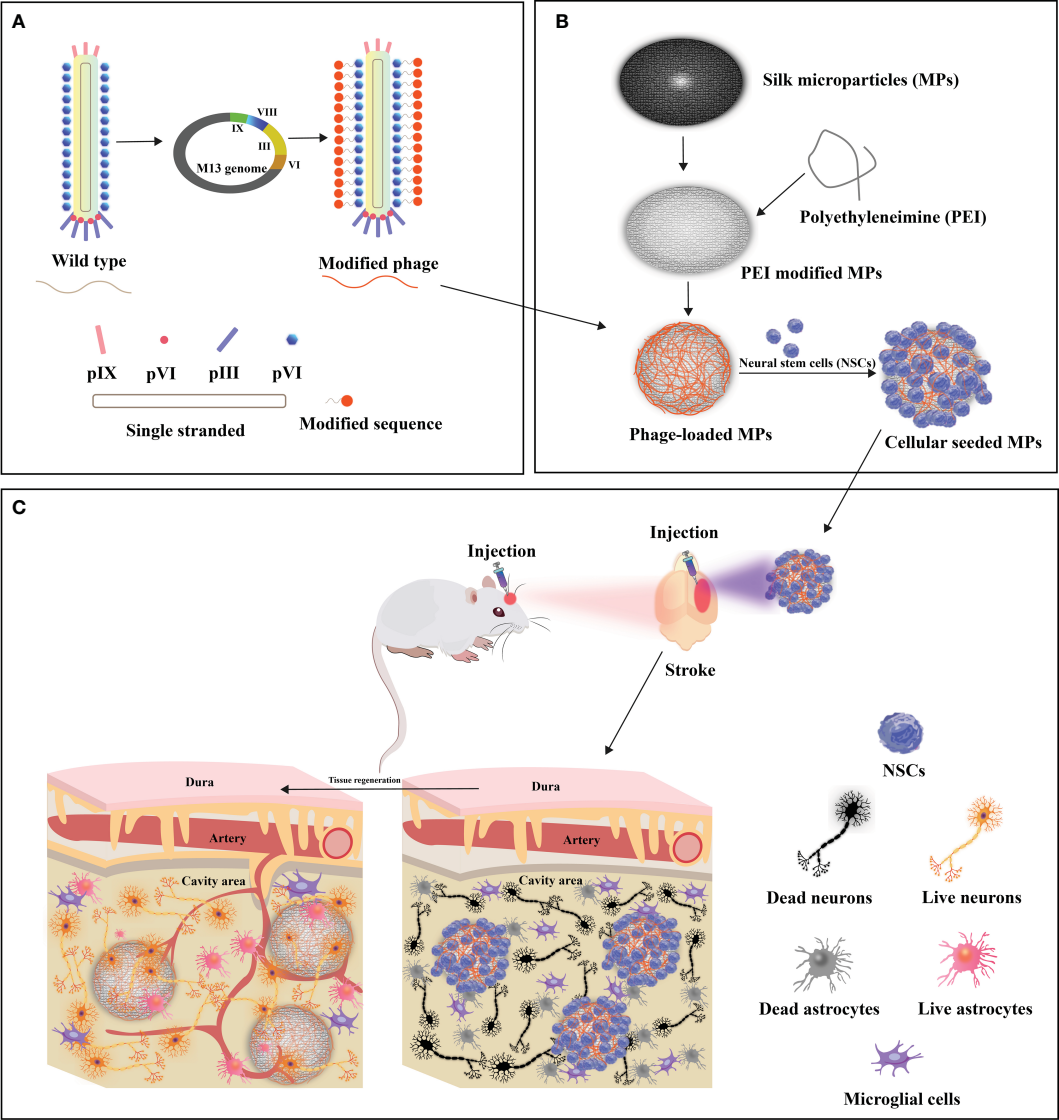
Design of functionalized filamentous phages-loaded microparticles(MPs) for post-stroke brain tissue repair. **(A)** Genetic linkage of modifying peptides to the N-terminus of the predominant capsid protein (pVIII) of WT-phage engenders the creation of modified phages. **(B)** Polyethyleneimine (PEI)-modified MPs undergo electrostatic adsorption with modified phages, thereby engendering phage-loaded MPs. Neural stem cells (NSCs) are subsequently implanted onto the surface of modified phage-MPs, and the cellular-seeded MPs are injected into the stroke-affected sites within rat brains. **(C)** The modified phage-MPs serve as physical scaffolds, fostering NSC proliferation and infiltration within the stroke cavity. The functionalized bacteriophage-loaded microparticles significantly amplify brain tissue repair in post-stroke injuries. Adapted from (26).

**Figure 6:**
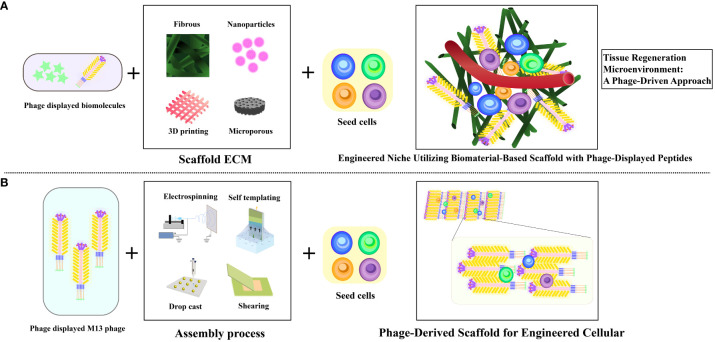
Illustrating diverse artificial cellular niches utilizing M13 phage as bionanomaterial. **(A)** Visualization of traditional biomaterial-infused scaffold featuring phage-displayed peptides and implanted stem cells to engineer a niche. **(B)** Depiction of biomimetic phage-infused scaffold encompassing implanted stem cells to engineer a niche. Adapted from (26).

Leveraging inherent regenerative capability within the human body, in conjunction with bio-nanomaterials, in a process termed *in situ* tissue regeneration, holds the promise of achieving tissue rejuvenation at its native location (75). Capitalizing on the versatile attributes of filamentous bacteriophages, encompassing neuronal protection, edema alleviation, infarct size reduction, and neurological improvement, opens avenues to integrate the mechanisms of *in situ* tissue regeneration into enhancing stroke recovery (26) (**Figure 5**). Currently, the therapeutic potential of extracellular matrix-mimicking scaffolds faces challenges associated with the incorporation of biomolecules. These challenges encompass the cost-effective production of bioactive agents and the intricate integration of these agents into scaffolds to maintain their bioactivity (76). Furthermore, the limitations in manipulating the fate of stem cells using chemically tethered peptides on biomaterial substrates could impede optimal outcomes (77). In contrast, the genetic incorporation of peptides onto M13 phage coat proteins offers an efficient and precise avenue, facilitating the seamless integration of biomolecules (78). Consequently, M13 phage emerges as a promising contender in the domain of tissue regenerative materials, leveraging its genetic control and modularity attributes to drive advancements in this field. These encompass its ability to precisely present functional peptides as signaling moieties on its surface (30, 33), its capacity to influence the behavior of macroscopic cells (79), its autonomous assembly into diverse structural configurations leading to varying topological effects (80), and its intrinsic biocompatibility and biodegradability (81). This amalgamation of attributes positions M13 phage as an exceptional choice for bionanomaterials, particularly in engineering artificial cellular “niches” (82) (**Figure 6**).

Furthermore, through its integration with bioactive compounds, therapeutic cells, and polymer solutions during the electrospinning process, M13 phage transforms into electrospun nanofibers that replicate the characteristics of the extracellular matrix (ECM), facilitating the seamless incorporation of molecular constituents with heightened efficiency (83). Notably, it is worth mentioning that certain studies have employed M13 phage to display functional peptides and integrate them into complementary scaffolds, rather than using it as a primary element for constructing tissue scaffolds (84) (**Figure 6**). M13 phages leverage electrospinning to create bionanofibers, ideal for tissue engineering (85–89). In Shin et al.’s study, nanofiber matrices merged PLGA with RGD-displaying M13 phages, improving fibroblast attachment and proliferation. Electrospun nanofibers, as seen in research on rat hippocampus-derived neural stem/progenitor cells (rNSCs), finely modulate cell behavior. These fibers mimic the extracellular matrix, promoting wound healing processes and cellular differentiation. Additionally, nanofibrous architectures have been explored for astrocyte differentiation (90–94). Zhou et al. have unveiled an original nanoridge-in-microridge (NiM) structure through a unique dip-pulling technique, akin to window blinds. These microridges, formed by parallel phage bundles, induced rapid stem cell differentiation into neurons and astrocytes within 8 days. With versatile applications in tissue regeneration, materials templating, sensing, and electrodes, NiM structures, adorned with diverse peptides on various substrates, hold immense potential across technological domains (95, 96). This pioneering approach leverages NiM structures’ multifunctionality and balance of electrostatic forces for various applications (96). Vascular repair and regeneration.

The accumulation of metabolic byproducts within blood vessels often culminates in obstruction, hampering smooth blood circulation and culminating in vascular occlusion, thus fostering cerebral ischemia and hypoxia, a precursor to stroke incidence. Hence, the post-stroke recuperation is significantly contingent on the repair and regeneration of blood vessels. The formation of neovascularization in ischemic tissues stands pivotal in realizing satisfactory tissue restoration following ischemic insults (97). Employing genetically engineered M13 phage offers an avenue to target tissue repair with precision, unraveling the prospects of instigating vascular tissue regeneration, thereby aligning with the objectives of vascular tissue restoration. For instance, the engineered M13 phage with nanofiber arginine-glycine-aspartic acid (RGD) showcases both angiogenic and antioxidative attributes, surmounting pathological milieus and bolstering the implantation of cells and adjacent soft tissues at ischemic sites. Through elevating the survival rate of endothelial progenitor cells (EPCs) implanted in ischemic murine models, this construct efficaciously curtails myogenic degeneration and fibrosis, while amplifying angiogenesis by harnessing the polarization of M2 macrophages (23). Furthermore, an novel approach involves the utilization of M13 phage nanofibers featuring high-density RGD peptides for populating 3D-printed bio-ceramic scaffolds. By seeding mesenchymal stem cells (MSCs) onto these phage nanofibers and subsequently implanting them into bone defects, the cascade of events ensues whereby MSCs are prompted to endothelialize and differentiate into osteogenic lineages, thereby instigating osteogenesis and vascular genesis (98).

### Neural repair and regeneration

Within the realm of Central Nervous System (CNS) Neural Regeneration, the restoration of axonal growth from emerging neural cells following traumatic brain injury is a paramount objective (99). Particularly in neurological conditions like cerebral stroke, the autophagy-mediated degradation of compromised tissue leads to the degradation of the Extracellular Matrix, thereby hindering the regenerative potential of brain tissue (100, 101). Leveraging delivery mechanisms such as M13 phages, therapeutic cells can be transported, and functional peptides carrying biochemical cues can be presented on the nanostructured virion, thereby creating an ECM-resembling scaffold or interfacing with compatible scaffolds effectively. Merzlyak and colleagues ingeniously engineered M13 phages to display neural cell-adhesive RGD or IKVAV peptides on the pVIII protein, orchestrating their assembly into intricate three-dimensional nanofiber matrices. This inventive approach concurrently incorporated neural progenitor cells (NPCs), fostering both cellular proliferation and differentiation, representing a promising strategy for neural tissue engineering (87). The results show that within seven days, adult rat neural progenitor cells (NPCs) exhibited a remarkable inclination to align along the M13 phage nanofibers, undergoing neural differentiation and extending neurites that remarkably mimicked the longitudinal axis of the phage fibers (87). This transformation orchestrated virion self-assembly into matrices with anisotropic topography, proficiently promoting neural progenitor cell (NPC) growth. The inclusion of biochemical and topographical cues within the RGD-phage films significantly increased NPC proliferation in comparison to the control group residing in wild-type-phage films. Most significantly, differentiating NPC neurites exhibited a strikingly accelerated growth rate, extending lengths four to seven times greater by the fourth day (23). Zhou et al. extended the paradigm to include not only neurons but also the crucial involvement of astrocytes in cerebral development. Astrocytes play a pivotal role in brain assembly and the growth of extracellular matrix constituents, including fibronectin and laminin (96). Their study explored the dual differentiation potential of human induced pluripotent stem cell (hiPSC)-derived neural progenitor cells (NPCs) into both neurons and astrocytes. They utilized a Nanofiber-mediated (NiM) construct ingeniously composed of M13 phage (96). The orchestrated manipulation of topographical influences, synergistically ameliorated by the inclusion of RGD peptides, has demonstrated a significant impact on NPCs. This effect is marked by the expression of neuron-specific markers and the accelerated generation of astrocytes within a remarkably short timeframe of just eight days, in contrast to the conventional approach of employing an astrocyte-inducing medium, which typically requires around 30 days to achieve similar outcomes (102).

The restoration of optimal functionality within compromised cerebral tissue is a complex process that relies on the establishment of intricate vascular networks. In the context of stroke potential therapy, Liu and colleagues conducted pioneering research on the potential of the M13 phage to promote dual neurogenesis and angiogenesis after cerebral infarction (26). Their approach involved genetically incorporating high-density RGD peptides onto the pVIII protein of the M13 phage, creating the customized “R-phage.” Simultaneously, silk fibroin microparticles (MPs) were crafted from silkworm cocoons, ensuring biocompatibility, and coated with polyethyleneimine (PEI) to enable the electrostatic attachment of R-phages onto the MPs. The strategic transplantation of NSC-seeded R-phage-MPs into ischemic regions within rat brains initiated a complex cascade of reparative events, ultimately enhancing brain tissue recovery post-stroke. This process resulted in the augmented differentiation of neural stem cells (NSCs), leading to the formation of densely interconnected, axon-rich neurons and an improvement in motor function. Moreover, this interplay stimulated a remarkable surge in revascularization and angiogenesis orchestrated through endothelial cell activation. This orchestrated response heighten nutrient and oxygen delivery to the growing neuronal environment, facilitating robust neurogenesis.

### Progressing bacteriophage therapy for stroke recovery

In the realm of our investigation, significant and noteworthy advancements have emerged. For instance, the work by Xiangyu Liu and colleagues (26) has unveiled a groundbreaking development. Through genetic engineering, they have restructured filamentous phages to yield a novel biogenic material known as R-phage-MPs, specifically designed for regenerating cerebral tissue within the confines of stroke-induced cavities. This study underscores the material’s remarkable capacity to stimulate the regeneration of vascular and neural networks in stroke-affected regions, concomitantly mitigating the scar formation of astrocytes and the aberrant proliferation of neural progenitor cells. These findings present an effective therapeutic strategy for post-stroke neural recovery. Furthermore, this research has shed light on a novel approach to crafting biogenic materials for brain tissue regeneration, achieved through genetic means, allowing virtually any peptide to be displayed on the surface of bacteriophages. In a different line of pioneering investigations, exemplified by the work of Zhou, Li et al. (81), they introduced a novel concept, the Nanoridge-in-Microridge (NiM) architecture, derived from filamentous M13 bacteriophages. This revolutionary structural design holds the potential to address neurodegenerative diseases (NDD), offering fresh perspectives and therapeutic insights. Studies have indicated that NiM structures can be instrumental in the treatment of NDD. Furthermore, these structures enable the establishment of co-culture models involving neurons and astrocytes, facilitating the exploration of their intricate interactions. This, in turn, provides invaluable insights into potential NDD therapies. Additionally, Mao’s laboratory has explored the use of bacteriophages and peptides as biofunctional materials, as demonstrated in the work of (103). Using bacteriophage display techniques, they successfully fused gene sequences from two distinct peptides. One peptide segment binds to Bombyx mori silk fibroin membranes, while the other originates from bone growth factors. This novel chimeric peptide construct exhibits elevated osteogenic efficacy. Their pioneering work offers a universal strategy for combining different peptide segments to innovate approaches in the functionalization of biomaterials.

Furthermore, our research domain has witnessed the emergence of several significant review articles, providing comprehensive insights into the latest advancements. Notably, the work of Cheng Chang and colleagues (48) stands out. In their publication, they highlight the unique attributes of M13 bacteriophages as novel biogenic nanomaterials, rich with a diverse array of biological molecules. This seminal review delves into cutting-edge research and developments in the field of nanomedicine, with a particular focus on the utilization of M13 bacteriophages as therapeutic agents. In summary, bacteriophage therapy presents new opportunities for promoting stroke recovery. While there are currently no clinical cases using bacteriophage therapy for stroke treatment, we believe that with advancements in scientific technology and further research, this approach has the potential to become a therapeutic option in the field of stroke treatment (104), significantly improving patients’ health and quality of life. Nevertheless, continuous exploration and relentless efforts are still required.

## Confronting dilemmas and challenges in stroke potential therapy

Stroke treatments, including pharmacological, interventional, and surgical methods, have shown limitations and side effects, often insufficient in fully restoring neural function. This drives the need to explore novel treatment modalities. Emerging biotechnological therapies such as stem cell, gene, and neurostimulation approaches aim to restore neural cells, facilitating cerebral tissue regeneration and functional recovery (105). These therapies offer advantages like precision and minimal invasiveness but are faced with challenges in safety and efficacy. Current stroke treatments, primarily reliant on acute measures, also possess limitations that impede the restoration of cerebral tissue.

### Tissue regeneration

Stroke, an acute cerebrovascular disorder, results from the abrupt occlusion or rupture of cerebral blood vessels, leading to localized cerebral ischemia or hemorrhage and consequential focal brain injury (5). Post-stroke, the brain initiates a complex process of tissue repair and functional recovery. In the early stages, damaged neural cells release inflammatory cytokines, triggering a response that plays a crucial role in processes like cell apoptosis, necrosis, and the recruitment of immune cells, potentially worsening neural damage (106). Over the subsequent weeks post-stroke, tissue repair commences. Activated neural stem cells and progenitor cells migrate to the affected region, with their unique ability to proliferate and differentiate into various neural cell types, playing a pivotal role in the recovery process (107). Simultaneously, neovascularization takes place, restoring blood supply to the affected area, delivering vital oxygen and nutrients required for cerebral tissue rehabilitation (108). However, the journey of post-stroke tissue repair often faces challenges. Excessive inflammatory responses and reduced neural stem cell functionality can impede achieving optimal recovery outcomes. Consequently, there’s an active exploration of strategies to intervene and improve the post-stroke tissue regeneration process. Notably, challenges identified in previous studies demand a targeted research focus to overcome these issues. Specifically, studies aimed at investigating the modulation of inflammatory responses, enhancing neural stem cell functionality, and identifying novel approaches to facilitate post-stroke tissue regeneration are imperative. One significant area demanding further exploration is the thorough evaluation of bacteriophage-mediated mechanisms for promoting cerebral tissue repair after a stroke. This avenue holds substantial potential for advancing the field of post-stroke recovery.

### Challenges in bacteriophage therapy for stroke recovery

Bacteriophage therapy for stroke has demonstrated promising outcomes in preclinical studies. Bacteriophages’ unique structure enables the fusion of exogenous peptides to their coat protein N-termini, achieving therapeutic objectives by presenting signaling peptides on the phage surface (109). Research indicates minimal adverse reactions and no observed side effects or detrimental brain tissue impairment upon introducing bacteriophages (110). Furthermore, phage display techniques allow the screening of target peptides and the synthesis of monoclonal antibodies with unique mechanisms of action. However, bacteriophage therapy faces significant limitations that demand further investigation. The potential demonstrated in preclinical studies necessitates comprehensive validation of its efficacy within the human body. Addressing this requires additional clinical trials that align therapeutic safety and efficacy findings from animal models with actual clinical settings. Overcoming the challenge of bypassing the blood-brain barrier in bacteriophage therapy remains crucial to ensure effective delivery to the damaged brain region. Additionally, the stability, persistence, and optimization of phage display technology warrant further exploration to optimize targeting efficiency (16). Advancements in molecular biology and genomics provide opportunities for furthering bacteriophage therapy. Tailoring bacteriophages for precise interventions using gene editing techniques is one such promising approach for stroke recovery. Reducing neuroinflammation is pivotal, necessitating research to optimize the therapy’s efficacy post-stroke (111). Moreover, combining bacteriophages with interventions like stem cell therapy and pharmacotherapy could lead to more potent treatment strategies. Innovation in monoclonal antibodies through phage display-driven screening could effectively address existing resistance concerns (112). As the field of molecular biology and genomics progresses, comprehensive research, thorough clinical trials, and technological advancements will propel phage therapy as a pioneering approach in enhancing stroke recovery (106).

## Safety evaluation in bacteriophage therapy

The resurgence of bacteriophage therapy in disease treatment has highlighted its efficacy in recent years. However, due to its biological nature, bacteriophage formulations, production procedures, and treatment methods substantially differ from traditional pharmaceutical approaches. This distinction calls for a comprehensive safety evaluation. Existing safety assessment systems, primarily tailored for conventional antimicrobial drugs, lack sufficient specificity for bacteriophage formulations, especially those of genetically engineered variants. As a result, the establishment of a dedicated safety assessment protocol tailored to bacteriophage therapy is imperative (113). Safety evaluations should encompass a broad spectrum of assessments, including biological characteristics, toxicity, immunogenicity, and pathogenicity specific to genetically engineered bacteriophages (113, 114). Ethical considerations, patient autonomy, and compliance with legal regulations during production are crucial to ensuring patient safety and ethical standards (113, 115, 116). Conducting extensive safety assessments is indispensable before the clinical implementation of pioneering treatments, such as genetically engineered bacteriophages in stroke potential therapy (113). Essential components of patient care in this realm involve regular radiological examinations and an emphasis on psychological well-being (117–119). These practices are pivotal in ensuring comprehensive patient safety and holistic care in the context of bacteriophage therapy potential for enhancing stroke recovery.

## Advancing stroke therapies to clinical application

To effectively transition the concept of novel stroke therapies to clinical practice, several crucial studies must be conducted. The current paradigm of stroke treatment faces limitations in fully restoring cerebral tissue and functional recovery. The pressing need for pioneering therapies, such as stem cell, gene, and neurostimulation approaches, to achieve more precise and minimally invasive interventions warrants rigorous examination. Clinical trials are imperative to assess the safety and efficacy of these emerging therapies in stroke treatment. Conducting comprehensive studies that move beyond preclinical findings to verify the feasibility of these therapies within the human body is essential. Kuriakose & Xiao (105) underscore the need for stringent clinical assessments to bridge the gap between laboratory research and real-world applicability. Long-term safety monitoring is critical to ascertain the enduring safety profile and effectiveness of these novel therapies over extended periods (105). Post-trial assessments are crucial to evaluate the sustained impact and side-effect profiles in real-world scenarios (120).

Comparative studies against existing acute measures in stroke treatment are vital to delineate the relative benefits and limitations of these pioneering approaches. Comparative analysis will provide a clearer understanding of the advantages and drawbacks of the emerging therapies compared to conventional treatment modalities (121). In-depth studies elucidating the underlying mechanisms of action of these novel therapies are essential to ensure targeted and effective interventions. Understanding the specific interactions and pathways involved in neural regeneration and tissue repair will guide the development of more precise and effective treatments (122). Evaluation of patient-centric outcomes and quality of life assessments after the application of these novel therapies is necessary. Analyzing the impact on patients’ quality of life, functional recovery, and long-term rehabilitation is vital to assess the true clinical significance of these pioneering approaches (105). Moving forward, these comprehensive studies are critical to establish the safety, efficacy, and practical applicability of the novel stroke therapies in real-world clinical settings, ensuring a transformative shift in stroke treatment modalities.

## Conclusion

Stroke represents a neurological impairment resulting from acute focal injury within the central nervous system due to vascular causes, often leading to functional deficits in specific bodily regions (5). Conventional therapeutic approaches predominantly involve thrombolysis, surgical intervention, and postoperative physical rehabilitation. However, these methods are constrained by limitations and potential side effects, rendering them insufficient to substantially mitigate disability. Phage therapy introduces a novel avenue for enhancing stroke recovery (104, 123). Patients facing diseases lacking effective cures are increasingly exploring bacteriophage therapy, even driving medical tourism. Industrial development also continues to embrace bacteriophages (124). Reevaluating the applications of engineered bacteriophages reveals novel possibilities in enhancing stroke recovery and brain regenerative biomaterials (26). The nanofiber structure and genetic modifications of filamentous bacteriophages show promise as potential biomaterials for addressing complex brain tissue concerns. These nanofibers aid in enhancing neural stem cell activity and stimulating vascular generation at stroke sites, potentially enhancing stroke therapies. Genetic engineering techniques are rapidly advancing bacteriophage research (125). The distinctive architecture of bacteriophages enables integration of exogenous peptides onto their coat proteins, endowing these peptides with targeting attributes that bolster disease treatment (16). Employing phage display technology can uncover stroke-relevant targeting peptides, facilitating the development of monoclonal antibodies with unique mechanisms via targeted modifications. Furthermore, integrating peptides into the shell proteins of filamentous bacteriophages also contributes to facilitating the recovery process, reducing inflammatory responses and scar thickness of astrocytes, while fostering neuroblast reactions in the brain’s subventricular zone. Bacteriophage therapy faces limitations requiring more research. Preclinical studies’ potential needs validation in humans, requiring clinical trials aligning safety and efficacy outcomes. Overcoming challenges in blood-brain barrier penetration is crucial for effective brain delivery. Further exploration of bacteriophage display technology for stability and targeting is necessary. Establishing a safety assessment, covering biological traits, toxicity, and immunogenicity, is essential for genetically engineered bacteriophages.

## Author contributions

YL: Conceptualization, Writing – original draft, Writing – review & editing. K-DY: Conceptualization, Investigation, Writing – original draft. D-CK: Investigation, Writing – review & editing. X–ML: Investigation, Writing – review & editing. HD: Investigation, Software, Writing – review & editing. J-FY: Investigation, Software, Writing – review & editing.
